# Dietary modulation shapes gut microbiota and digestive physiology associated with specialized frugivory in *Teratoscincus roborowskii*

**DOI:** 10.3389/fmicb.2025.1675240

**Published:** 2026-01-08

**Authors:** Yi Yang, Huawei Feng, Ziyi Wang, Ruichen Wu, Yuhan Zheng, Xunheng Wu

**Affiliations:** 1 Xinjiang Key Laboratory for Ecological Adaptation and Evolution of Extreme Environment Organisms, College of Life Sciences, Xinjiang Agricultural University, Urumqi, China; 2 Xinjiang Agricultural University Wildlife Gut Microecology and Metabolism Research Center, College of Life Sciences, Xinjiang Agricultural University, Urumqi, China

**Keywords:** adaptive evolution, *Capparis spinosa*, dietary shifts, digestive physiology, frugivorous behavior, gut microbiota, *Teratoscincus roborowskii*

## Abstract

Diet plays a key role in determining the composition and function of the gut microbiota. *Teratoscincus roborowskii* inhabits extreme desert environments and displays a unique frugivorous behavior. To investigate the relationship between its gut microbiota and metabolites associated with this specialized diet, the gut microbiota and key metabolite variations in *T. roborowskii* fed varying proportions of *Capparis spinosa* (capers) were analyzed using 16S rRNA sequencing and metabolomic profiling. Our results showed that trypsin activity was significantly higher in the mealworms group than in the capers groups. In contrast, the lipase activity was the highest in the capers-80% group, and the α-amylase activity was the highest in the capers-50% group. These patterns indicate that digestive enzyme activity reflects dietary composition. In addition, *T. roborowskii* fed either mealworm or caper-based diets shared the same dominant microbiota at the phylum level. However, the inclusion of capers significantly increased the abundance of *Blautia* in *T. roborowskii*, which are enriched in folate biosynthesis and purine metabolism pathways, supporting the idea that changes in diet can rapidly reshape the gut microbiota. Notably, diet, gut microbiota, digestive enzymes, and fecal metabolites are closely linked and interact with each other, indicating that *T. roborowskii* can adapt to dietary changes by modulating its gut microbiota and digestive physiology. Our study offers valuable insights into the gut microbiota of desert lizards and warrants further exploration of the relationship between desert lizards and desert plants.

## Graphical abstract

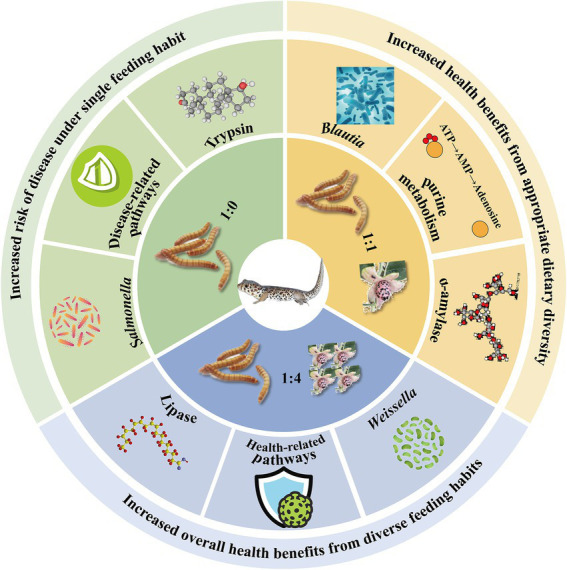

## 1 Introduction

Vertebrate digestive systems contain diverse and abundant microbial communities that provide valuable services to the host, including nutrition, immune regulation and developmental support (Ley et al., 2008; Lathrop et al., 2011). Numerous studies have revealed that gut microbial communities are influenced by various factors, including diet, habitat, evolutionary history, and sex (Kohl et al., 2016; Liu et al., 2024; Youngblut et al., 2019; Zhang et al., 2022). Host diet stands out as one of the most pervasive and influential environmental determinants of gut microbiota composition across vertebrates (Kostovcikova et al., 2019). The gut microbiota exhibits a high degree of plasticity and a rapid response ability, undergoing rapid and significant changes in response to short-term diet interventions. It has been shown that omnivorous lizards *Liolaemus ruibali*, which consume an average of only 16% of the plants in the wild, exhibited a more diverse gut microbiota, with significantly higher abundance of Melainabacteria and *Oscillospira* after feeding on a plant-rich diet (Kohl et al., 2016). Furthermore, dietary intake—particularly of nondigestible carbohydrates, proteins, and fats—strongly shapes microbial metabolite formation. This process is dictated primarily by the chemical structures of the substrates and the microbial pathways through which they are processed (Flint et al., 2015). These microbial transformations occur principally within the large intestine, where a dense microbial community drives significant metabolic activity. For instance, studies have shown that mice fed an animal protein-based diet increased the relative abundance of *Enterococcus*, *Streptococcus*, and *Peptostreptococcus*, which have been linked to gastrointestinal diseases. In contrast, mice fed plant protein-based diets increased the abundance of Lactobacillaceae, Lachnospiraceae, and Erysipelotrichaceae, which stimulate short-chain fatty acids (SCFAs) production (Kostovcikova et al., 2019).

To maximize net nutrient gain, a dietary shift should lead to changes in gut physiology to match the new diet, whether over short or long timescales. There should be a match between gut function (digestive enzyme activities and nutrient transport rates) and the food ingested by an animal (Karasov and Douglas, 2013). Different substrate types require specific complements of secretions and enzymes for their breakdown, as well as particular mechanisms for the absorption of their breakdown products. Some food types can be broken down relatively rapidly by typical enzymatic activities (disaccharidases, amylases, proteases, peptidases, lipases) that are endogenously present in the digestive tracts of most animals. However, plant cell walls or arthropod cuticle/chitin, which resist digestion by endogenous enzymes and therefore require microbial assistance in the breakdown of these substrates (Karasov et al., 2011). Studies on fish with different feeding habits (omnivorous, herbivorous, plankton-feeding, and carnivorous) have shown that the trypsin and amylase activities were significantly higher in carnivorous and herbivorous species, respectively, and *Acinetobacter*, which might help the host digest protein, and *Bacteroides*, which might help the host digest cellulose, were the most dominant flora in carnivorous and herbivorous fishes, respectively (Jiao et al., 2023).

Frugivory is considered an important feeding behavior among various animals, particularly within specific ecosystems. Frugivores obtain nutrition by consuming fruits and play a key role in the dispersal of these fruits. This behavior not only affects the nutrition and health of the animals themselves but also has a profound impact on plant reproduction and ecosystem diversity (Yang et al., 2021). Lizard frugivory has been reported in a variety of environments, including Mediterranean type climate ecosystems, temperate rainforests and high-elevation Andes shrubs. The dispersal of seeds by lizards has been described as a typical island phenomenon (Olesen and Valido, 2003). Therefore, despite being relatively rare among lizards, frugivory may have significant implications for gut microbiota composition.

*Teratoscincus roborowskii* is an endemic species that is only distributed in the Turpan Depression of the Xinjiang Uyghur Autonomous Region, China (Wang et al., 2024a, 2024b). The investigation of *T. roborowskii* has mainly focused on behavior, ecology, and morphology, encompassing mimicry (Autumn and Han, 1989), foraging modes (Werner et al., 1997), activity rhythm (Song et al., 2017), sexual dimorphism, diet, skeletochronology (Li et al., 2010), home range (Li et al., 2013), habitat (Song et al., 2017), seed dispersal (Yang et al., 2021), and the digestive tract morphology (Wang et al., 2024a,b). Field observation and dietary analysis have shown that the dietary habits of *T. roborowskii* display a significant seasonal shift. Our previous study showed that 85% of the total biomass consumed by *T. roborowskii* was *Capparis spinosa* (capers), suggesting that capers may play a crucial role in the adaptation of *T. roborowskii* to the extremely arid environment in the Turpan Basin (Yang et al., 2021). *T. roborowskii*’s main food sources are insects in spring, whereas they eat a lot of capers in summer and autumn, which lead to seasonal shifts in gut microbiota and metabolites (Gao et al., 2023). Additionally, *T. roborowskii* has been demonstrated as an effective disperser of capers seeds, enhancing their germination rates (Yang et al., 2021). Due to the special frugivorous behavior of *T. roborowskii*, we addressed three key questions: (1) Is there a mutually beneficial relationship between *T. roborowskii* and capers? (2) Does *T. roborowskii* adapt to the dietary shift by reshaping its gut microbiota? (3) Does *T. roborowskii* adapt to different proportions of capers by modulating its digestive physiology?

Here, combining the high consumption of capers by *T. roborowskii* in the wild, capers with varying contents (50 and 80%) were added to its diet, the 16S rRNA and metabolomics using liquid chromatography mass spectrometry (LC–MS) were used to compare the differences of diet, composition and its key metabolite of gut microbiota and digestive function of the *T. roborowskii* that feeding with different proportions of capers fruit. To elucidate the effects of variation in diet compositions on the structure, metabolites and digestive physiology, and further explore correlations between gut microbiota and its metabolites associated with the special frugivorous behavior of *T. roborowskii*.

## 2 Materials and methods

### 2.1 Animal and feces collection

**Figure 1:**
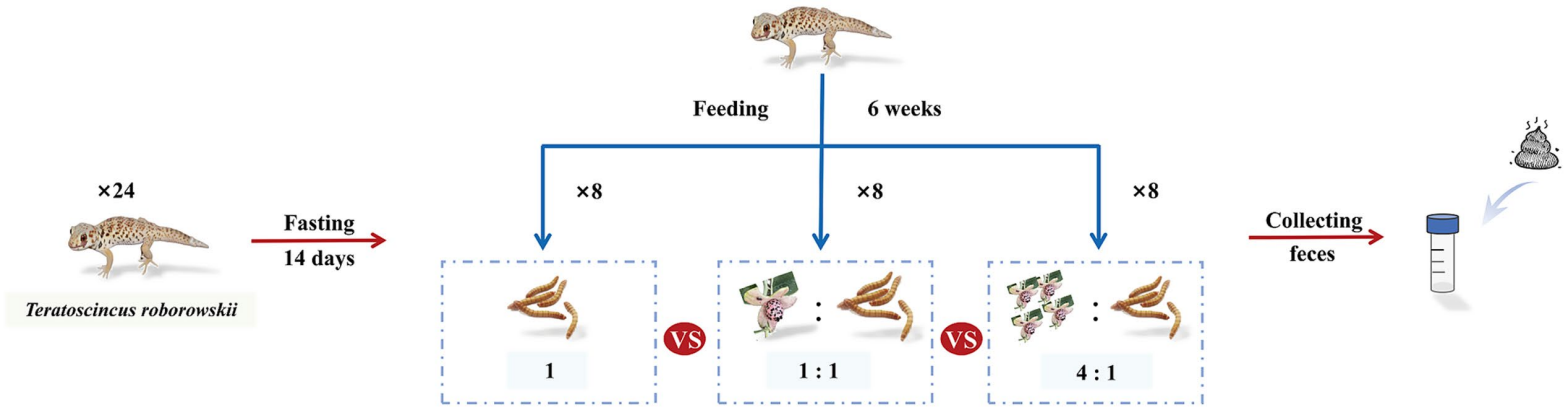
Test grouping and treatment in *T. roborowskii* fed mealworm or capers-based diets.

Twenty-four *T. roborowskii* were all adult and captured in May 2024 at the Turpan region, which is located in the Turpan Basin in the Xinjiang Uyghur Autonomous Region of China (E89°11′, N42°54′). All adult lizards were placed in 30 × 21 × 15.5 cm (L × W × H) plastic feeding boxes. All lizards were fasted for 14 days (Yang et al., 2021), aiming to empty the *T. roborowskii* intestine of residues and ensure accuracy of feeding experiments afterwards. All *T. roborowskii* were divided into capers-50% group, capers-80% group and mealworms group, with 8 lizards in each group. During captivity, the lizards in mealworms group were fed with 3–4 live mealworms. The lizards in capers group were fed with mealworms and capers, which were thoroughly mixed with a homogenizer at a ratio of 1:1 and 1:4. Fecal samples were collected after 6 weeks. The dry matter, organic matter, fat, coarse fiber, crude protein, calcium, and phosphorus of the three diets were determined according to the AOAC (2019) 930.15, 942.05, 954.02, 962.09, 984.13, 968.08, and 965.17 methods, respectively. Energy was determined by direct combustion in an adiabatic bomb calorimeter. We monitored fecal excretion every 3 h to ensure only fresh samples were collected. All the fecal samples were collected into sterile cryovials using sterilized tweezers and were snap-frozen in liquid nitrogen immediately; then, they were stored at −80 °C (Figure 1). All the experimental procedures involving animals were approved by the Animal Welfare and Ethics Committee of Xinjiang Agricultural University, Urumqi, Xinjiang, China. After the experiment was completed, all the lizards were released in their original habitats.

### 2.2 Assessment of digestive enzyme activity

#### 2.2.1 Assessment of trypsin activity

The trypsin assay kit (Geruisi, Suzhou, China; Cat. No. G1209F) was used for this assay. Trypsin catalyzes the hydrolysis of N-benzoyl-DL-arginine-p-nitroaniline hydrochloric acid (BAPNA) to p-nitroaniline, which has a maximum absorption peak at 405 nm. The activity of trypsin can be determined by measuring the increase rate of absorbance value. Experimental procedures: approximately 0.1 g of fecal sample was added to 1 mL of normal saline and homogenized in an ice bath. The samples were centrifuged at 4 °C × 12,000 rpm for 10 min, leaving the supernatant to be measured. The colorimetric tank of the spectrophotometer was preheated at 37 °C in advance. The timing was started while the supernatant was mixed with the reaction liquid (BAPNA) and then poured into a cuvette (1 cm optical path). Absorbance values were measured and recorded at 405 nm and again 10 min later. 1 nmol of p-nitroaniline is produced per gram of tissue per minute as an enzyme activity (nmol/min/g).

#### 2.2.2 Assessment of lipase activity

The lipase assay kit (Geruisi, Suzhou, China; Cat. No. G0902F) was used for this assay. Lipase hydrolyzes the substrate p-nitrophenol ester to produce p-nitrophenol with color. The lipase activity can be obtained by measuring the absorbance value at 405 nm wavelength. Experimental procedures: the sample solution was extracted using the same procedure as trypsin above. The timing was started while the supernatant was mixed with the reaction liquid (p-nitrophenol ester) and then poured into a cuvette (1 cm optical path). Absorbance values were measured at a wavelength of 405 nm and again after 10 min. The amount of enzyme releasing 1 nmol of p-nitrophenol per minute per gram of tissue was defined as one unit of enzyme activity (nmol/min/g).

#### 2.2.3 Assessment of α-amylase activity

The α-amylase assay kit (Geruisi, Suzhou, China; Cat. No. G0510F) was used for this assay. The reducing sugar produced by the hydrolysis of starch catalyzed by α-amylase can make 3,5-Dinitrosalicylic acid turn brown red to 3-amino-5-nitrosalicylic acid, which has an absorption peak at 540 nm, α-amylase activity was calculated by the rate of absorbance increase.

Experimental procedures: (1) sample preparation: about 0.2 g of feces was added to 1 mL of 95% ethanol and mixed in an ice bath, then placed at 4 °C for 10 min. The samples were centrifuged at 4 °C × 12,000 rpm for 5 min, and the supernatant was discarded to leave the precipitate. An additional 1 mL 80% ethanol was added to the precipitate and mixed, and the above procedure was repeated once. 1 mL of the extract was added to the precipitate, mixed and left at 4 °C for 10 min, followed by centrifugation at 4 °C × 12,000 rpm for 5 min, then leaving the supernatant to be measured. (2) Instrumental detection: the supernatant was bathed at 70 °C for 15 min to inactivate β-amylase. After cooling, it was mixed with starch substrate and incubated at 40 °C for 5 min. The sample solution added with distilled water was used as a control. Finally, the reaction solution (3,5-dinitrosalicylic acid) was added to the two tubes and placed in a 95 °C water bath. After cooling, the absorbance value was read at 540 nm. The catalytic production of 1 μg maltose per gram of tissue per minute is defined as one enzyme activity unit (μg/min/g).

#### 2.2.4 Assessment of cellulase activity

The cellulase assay kit (Geruisi, Suzhou, China; Cat. No. G0510F) was used for this assay. Cellulase hydrolyzes cellulose to produce reducing sugars, such as cellobiose and glucose, which can reduce 3,5-dinitrosalicylic acid (DNS) under alkaline conditions to produce a brownish-red amino compound. Cellulase activity was determined colorimetrically at 540 nm by measuring the reducing sugars produced. Experimental procedures: the sample solution was extracted using the same procedure as α-amylase above. The substrate solution (cellulose) was mixed with the sample solution as the assay tube, and the sample with buffer solution was used as the control tube. The two tubes were placed in a 37 °C water bath for 60 min. Then the reaction solution (3,5-dinitrosalicylic acid) was added, mixed and incubated at 95 °C for 5 min. The cooled solution was placed in a cuvette and the absorbance value was read at 540 nm. Catalytic production of 1 μg of reducing sugar per gram of tissue per hour was defined as one unit of enzyme activity (μg/h/g).

### 2.3 High-throughput 16S ribosomal RNA gene sequencing

Total genomic DNA was extracted from fecal samples using the TGuide S96 Magnetic Stool DNA Kit (Tiangen Biotech Co. Ltd., Beijing, China) according to manufacturer’s instructions. The quality and quantity of the extracted DNA were examined using electrophoresis on a 1.8% agarose gel, and DNA concentration and purity were determined with NanoDrop 2000 UV–Vis spectrophotometer (Thermo Scientific, Wilmington, United States). The full-length 16S rRNA genes were amplified with primer pairs 27F: AGRGTTTGATYNTGGCTCAG and 1492R: TASGGHTACCTTG TTASGACTT. Both the forward and reverse 16S primers were tailed with sample-specific PacBio barcode sequences to allow for multiplexed sequencing. We chose to use barcoded primers because this reduces chimera formation as compared to the alternative protocol in which primers are added in a second PCR reaction. The KOD One PCR Master Mix (TOYOBOLife Science) was used to perform 25 cycles of PCR amplification, with initial denaturation at 95 °C for 2 min, followed by 25 cycles of denaturation at 98 °C for 10 s, annealing at 55 °C for 30 s, and extension at 72 °C for 1 min 30 s, and a final step at 72 °C for 2 min. The total of PCR amplicons were purified with VAHTSTM DNA Clean Beads (Vazyme, Nanjing, China) and quantified using the Qubit dsDNA HS Assay Kit and Qubit 3.0 Fluorometer (Invitrogen, Thermo Fisher Scientific, Oregon, United States). After the individual quantification step, amplicons were pooled in equal amounts. SMRTbell libraries were prepared from the amplified DNA by SMRTbell Express Template Prep Kit 2.0 according to the manufacturer’s instructions (Pacific Biosciences). Purified SMRTbell libraries from the pooled and barcoded samples were sequenced on a PacBio Sequel II platform (Beijing Biomarker Technologies Co. Ltd., Beijing, China) using Sequel II binding kit 2.0.

### 2.4 Bioinformatic analysis

The bioinformatics analysis of this study was performed with the aid of the BMKCloud.^1^ The raw reads generated from sequencing were filtered and demultiplexed using the SMRT Link software (version 8.0) with the minPasses ≥ 5 and minPredictedAccuracy ≥ 0.9, in order to obtain the circular consensus sequencing (CCS) reads. Subsequently, the lima (version 1.7.0) was employed to assign the CCS sequences to the corresponding samples based on their barcodes. CCS reads containing no primers and those reads beyond the length range (1,200–1,650 bp) were discarded through the recognition of forward and reverse primers and quality filtering using the Cutadapt (Bolger et al., 2014) (version 2.7) quality control process. The UCHIME algorithm (v8.1) (Martin, 2011) was used in detecting and removing chimera sequences to obtain the clean reads. Sequences with similarity >97% were clustered into the same operational taxonomic unit (OTU) by USEARCH (Edgar, 2013) (v10.0), and the OTUs conuts less than 2 in all samples were filtered.

Taxonomy annotation of the OTUs was performed based on the Naive Bayes classifier in QIIME2 (Bolyen et al., 2019) using the SILVA database (Quast et al., 2013) (release 138.1) with a confidence threshold of 70%. Alpha was performed to identify the complexity of species diversity of each sample utilizing QIIME2 software and alpha index comparison among groups was computed by ANOVA. Beta diversity calculations were analyzed by principal coordinate analysis (PCoA) to assess the diversity in samples for species complexity, and based on bray-curtis distances. One-way analysis of variance was used to compare bacterial abundance and diversity. Linear discriminant analysis (LDA) coupled with effect size (LEfSe) was applied to evaluate the differentially abundant taxa. The Kyoto encyclopedia of genes and genomes (KEGG) pathway analysis of the OTUs was inferred using PICRUSt2. Analysis of function difference between groups was calculated by Welch’s *t*-test. The online platform BMKCloud^2^ was used to analyze the sequencing data.

### 2.5 LC–MS metabolomics detection

The LC–MS system for metabolomics analysis is composed of Waters Acquity I-Class PLUS ultra-high performance liquid tandem Waters Xevo G2-XS QTof high resolution mass spectrometer. The column used is purchased from Waters Acquity UPLC HSS T3 column (1.8 um 2.1 × 100 mm). Waters Xevo G2-XS QTof high resolution mass spectrometer can collect primary and secondary mass spectrometry data in MSe mode under the control of the acquisition software (MassLynx V4.2, Waters). In each data acquisition cycle, dual-channel data acquisition can be performed on both low collision energy and high collision energy at the same time. The low collision energy is off, the high collision energy range is 10 ~ 40 V, and the scanning frequency is 0.2 s for a mass spectrum. The parameters of the ESI ion source are as follows: capillary voltage: 2,500 V (positive ion mode) or −2,000 V (negative ion mode); cone voltage: 30 V; ion source temperature: 100 °C; desolvent gas temperature 500 °C; backflush gas flow rate: 50 L/h; desolventizing gas flow rate: 800 L/h. The raw data collected using MassLynx V4.2 are processed by Progenesis QI software for peak extraction, peak alignment and other data processing operations, based on the Progenesis QI software online METLIN database and Biomark’s self-built library for identification.

After normalizing the original peak area information with the total peak area, the follow-up analysis was performed. Principal component analysis and Spearman correlation analysis were used to judge the repeatability of the samples within group and the quality control samples. The identified compounds are searched for classification and pathway information in KEGG databases. According to the grouping information, calculate and compare the difference multiples, *T*-test was used to calculate the difference significance *p*-value of each compound. The R language package ropls was used to perform OPLS-DA modeling, and 200 times permutation tests were performed to verify the reliability of the model. The VIP value of the model was calculated using multiple cross-validation. The method of combining the difference multiple, the *p*-value and the VIP value of the OPLS-DA model was adopted to screen the differential metabolites. The screening criteria are FC > 1, *p* < 0.05 and VIP > 1. The differential metabolites of KEGG pathway enrichment significance were calculated using hypergeometric distribution test.

## 3 Results

### 3.1 Effects of dietary capers proportions on nutrition profiles and digestive enzyme activities in *Teratoscincus roborowskii*

**Figure 2:**
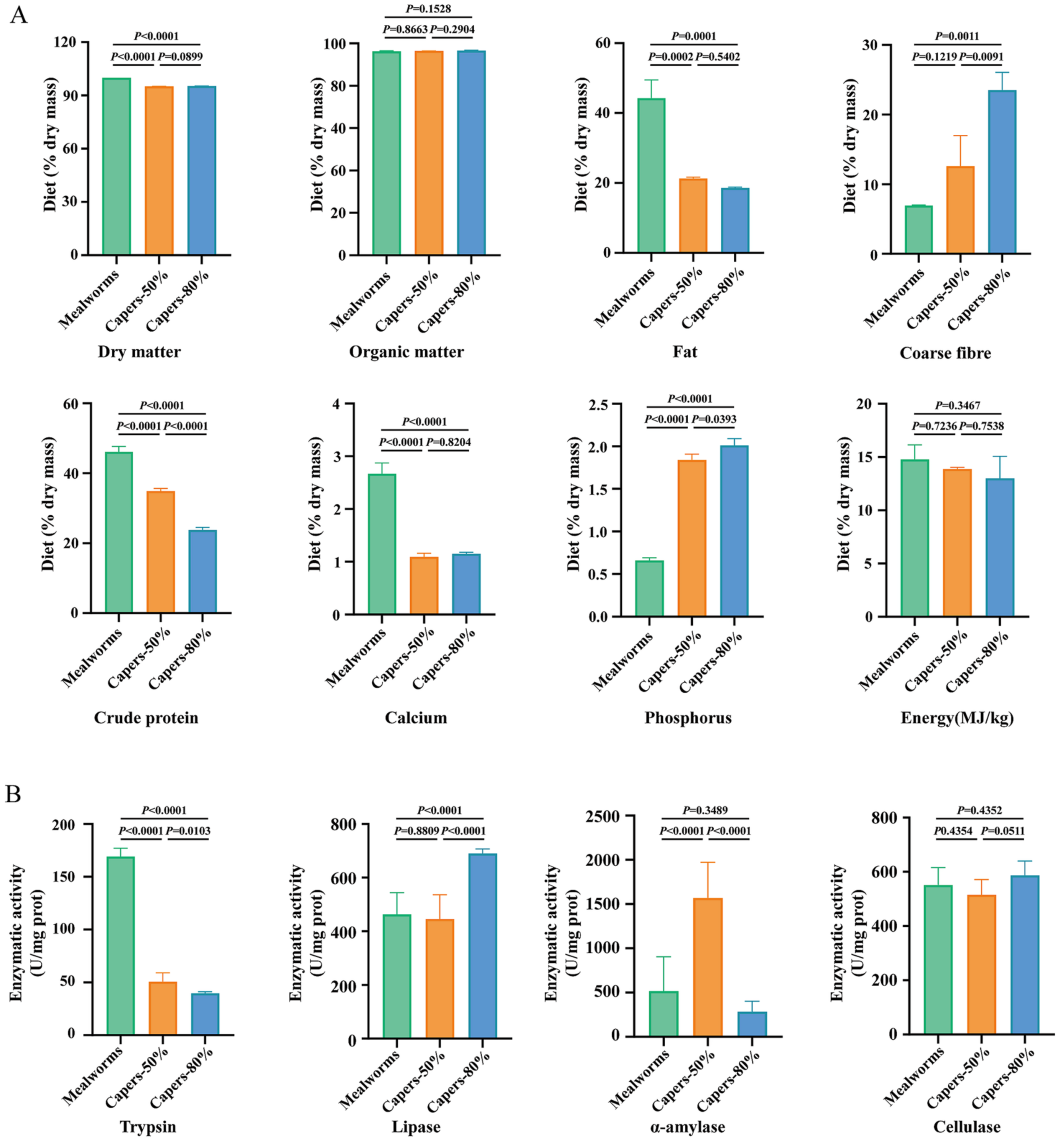
Dietary capers proportions on nutrition profiles and digestive enzyme activities in *T. roborowskii*. **(A)** Composition of diets fed to *T. roborowskii*. Composition is based on percent dry matter. **(B)** The digestive enzyme activities in *T. roborowskii* fed mealworm or capers-based diets. Significant differences in mealworms, capers-50% and capers-80% groups were tested by a two-tailed paired *t*-test, values of *p* < 0.05 were considered statistically significant.

Different diets result in distinct nutrient compositions in *T. roborowskii*. Dry matter, fat, crude protein, calcium, in the mealworms group were significantly higher than those in the capers groups. In contrast, coarse fiber and phosphorus in the capers-80% group were significantly higher than those in the mealworms group, while there was no significant difference in organic matter and energy among the three groups (Figure 2A). Digestive enzyme activities varied markedly among the dietary groups of *T. roborowskii*. The trypsin activity in the mealworms group was remarkably higher than that in the capers groups. The lipase activity in the capers-80% group were significantly higher than those in mealworms and capers-50% groups. The α-amylase activity in the capers-50% group were significantly higher than those in mealworms and capers-80% groups, while there was no significant difference in cellulase activity among the three groups (Figure 2B).

### 3.2 Analysis of gut microbiota diversity in *Teratoscincus roborowskii* fed mealworm or capers-based diets

**Figure 3:**
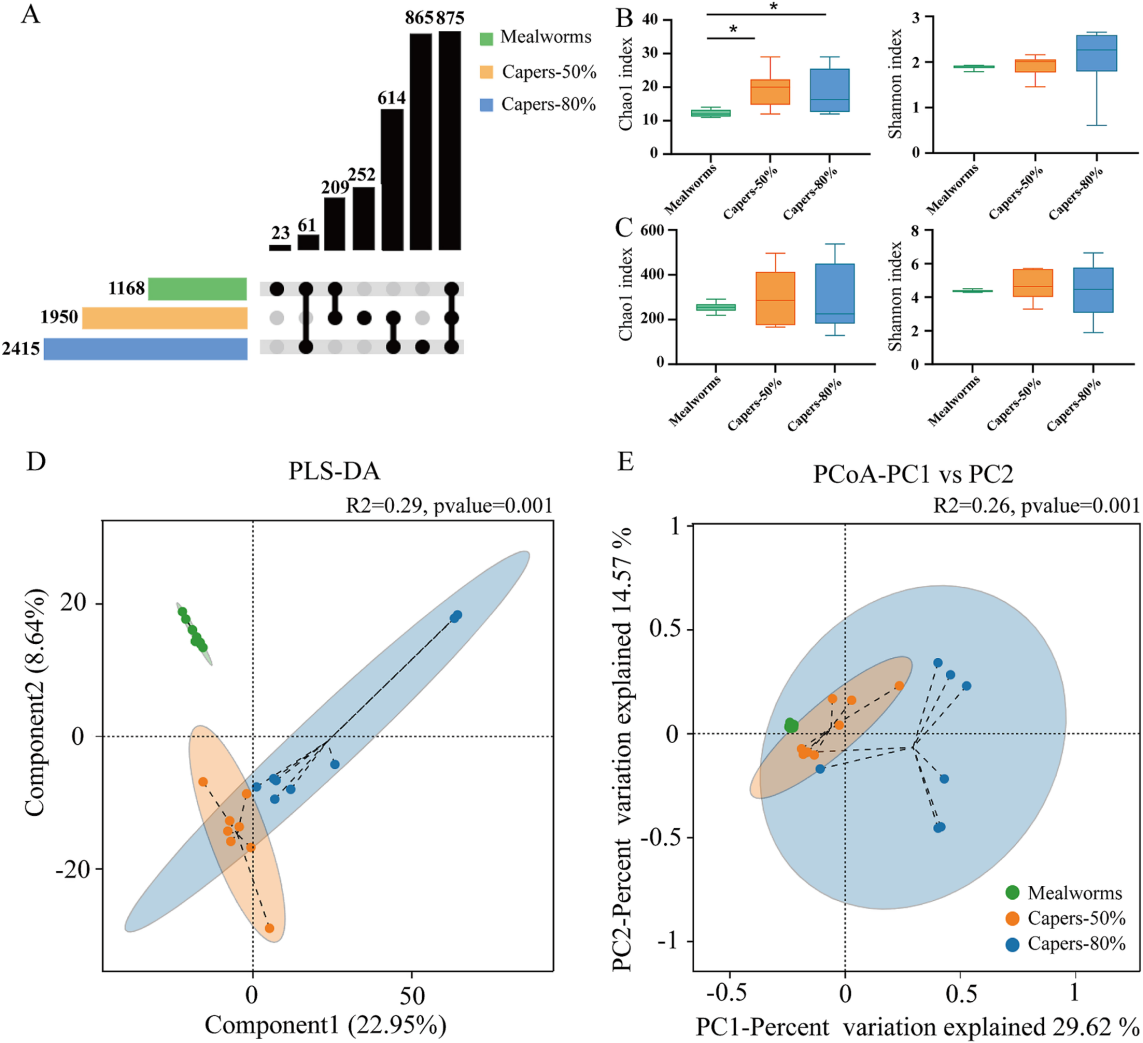
Microbiota diversity in *T. roborowskii* fed mealworm or capers-based diets. **(A)** UpSet plot of gut microbiota OTUs in *T. roborowskii* fed mealworm or capers-based diets. Alpha diversity index of gut microbiota at phylum **(B)** and genus **(C)** levels. PLS-DA **(D)** and PCoA **(E)** analysis of gut microbiota in *T. roborowskii* fed mealworm or capers-based diets. Alpha diversity was compared using Tukey-HSD multiple comparisons (*p < 0.05).

There were 2,415 OTUs in the capers-80% group, 1950 OTUs in the capers-50% group, and 1,168 OTUs in the mealworms group. A total of 875 OTUs were present in the three groups. The number of OTUs present in both the capers-80% and mealworms groups was 61, in both the capers-50% and mealworms groups was 209, and in both the capers-80% and capers-50% groups was 614. There were 23 unique OTUs in the mealworms group, 252 unique OTUs in the capers-50% group, and 865 unique OTUs in the capers-80% group (Figure 3A). There were no significant differences in alpha diversity among the three groups at the genus level. However, at the phylum level, the Chao 1 index in the capers-80% and capers-50% groups was significantly higher than that in the mealworms group (*p* < 0.05) (Figures 3B,C). The PLS-DA and PCoA plots (Figures 3D,E) revealed significant differences among the three groups. PCoA plots showed greater individual variability within the capers groups, suggesting that capers intake may reduce microbiota stability.

### 3.3 Gut microbiota composition and metabolic functional pathways in *Teratoscincus roborowskii* fed mealworm or capers-based diets

**Figure 4:**
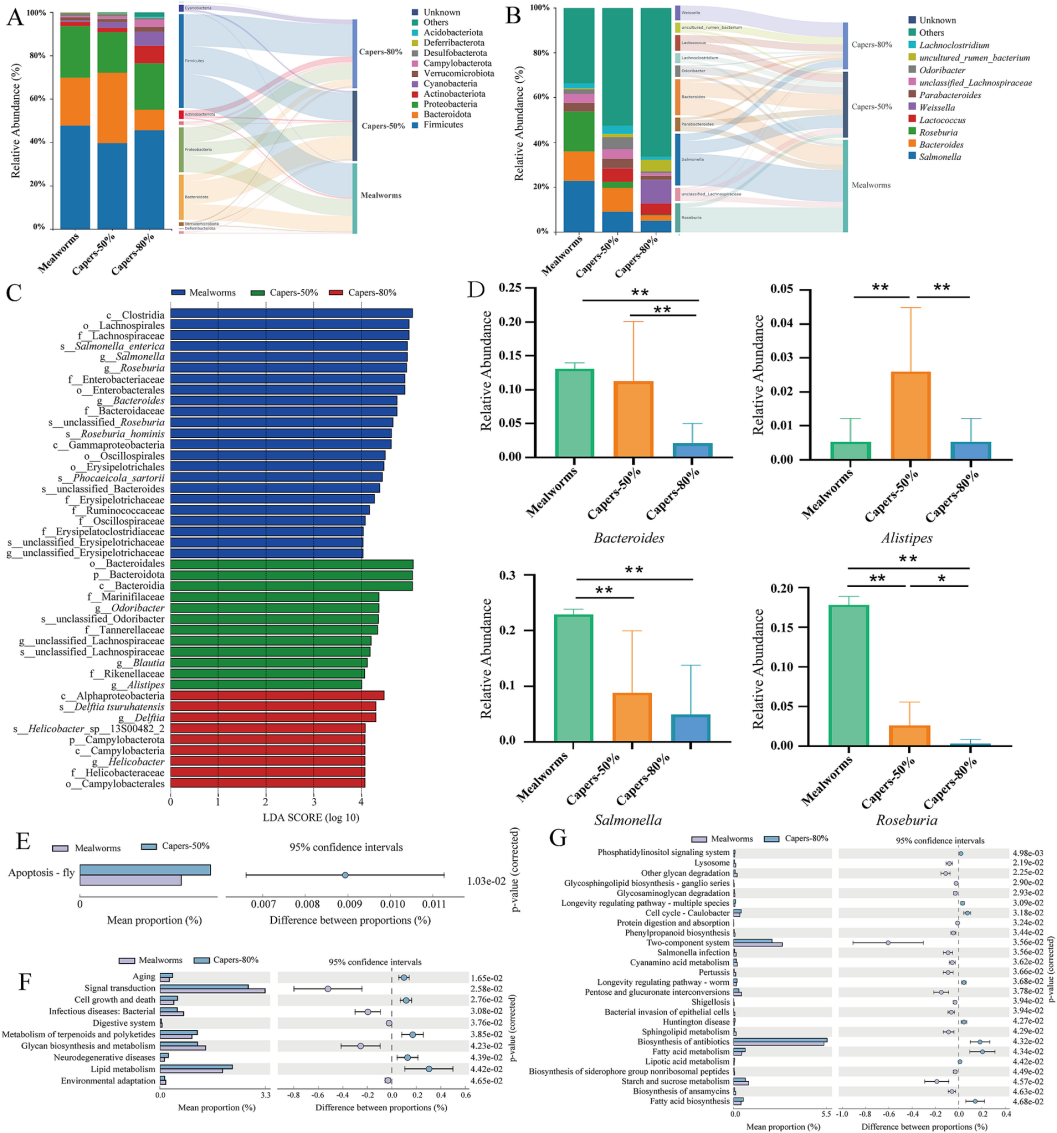
Composition and metabolic functional pathways of gut microbiota in *T. roborowskii* fed mealworm or capers-based diets. Relative abundance of gut microbiota in *T. roborowskii* fed mealworm or capers-based diets at phylum **(A)** and genus **(B)** levels. **(C)** LEfSe analysis of gut microbiota composition in *T. roborowskii* fed mealworm or capers-based diets (LDA > 4, *p* < 0.05). **(D)** Differential gut microbiota in *T. roborowskii* fed mealworm or capers-based diets, *p < 0.05; **p < 0.01. KEGG at the tertiary levels **(E)** between the mealworms group and capers-50% group. KEGG at the secondary **(F)** and tertiary levels **(G)** between the mealworms group and capers-80% group.

The proportions of gut microbiota under the different taxonomic classifications were shown in Figure 4. At the phylum level, the gut microbiota in the mealworms, capers-50% and capers-80% groups were mainly dominated by Firmicutes (47.86, 39.75, 45.68%), Bacteroidota (22.04, 32.50, 9.46%), and Proteobacteria (23.77, 18.63, 21.43%), while the other representative phyla included Actinobacteriota (1.95, 2.06, 8.09%) and Cyanobacteria (0.66, 2.61, 6.48%) (Figure 4A). At the genus level, the gut microbiota of *T. roborowskii* in the mealworms group were dominated by *Salmonella* (22.89%), *Roseburia* (17.80%), and *Bacteroides* (13.11%). Compared with the mealworms group, the dominant genera in the capers-50% group included *Bacteroides* (10.60%), *Salmonella* (9.13%), and *Lactococcus* (6.00%). In the capers-80% group, the dominant genera included *Weissella* (10.53%), *Salmonella* (5.18%), and *Lactococcus* (5.18%) (Figure 4B).

In addition, LEfSe analyses were performed between the three groups to estimate the difference in relative abundance at the different bacterial taxonomic levels (LDA > 4, *p* < 0.05) (Figure 4C). The relative abundances of Campylobacterota at the phylum level, *Helicobacter* and *Delftia* at the genus level in the capers-80% group were significantly higher than those in the mealworms and capers-50% groups. However, the relative abundances of Bacteroidota at the phylum level, *Odoribacter*, *Alistipes,* and *Blautia* at the genus level in the capers-50% group were significantly higher than those in the mealworms and capers-80% groups. The relative abundances of *Bacteroides*, *Roseburia*, and *Salmonella* at the genus level in the mealworms group were significantly higher than those in the capers-50% and capers-80% groups. The gut microbiota in the different groups of *T. roborowskii* showed different characteristics (Figure 4D). *Salmonella* in the mealworms group were significantly higher than those in the capers-50% and capers-80% groups. The *Bacteroides* in both mealworms and capers-50% groups were remarkably higher than those in the capers-80% group. The *Alistipes* in the capers-50% group were significantly higher than those in both mealworms and capers-80% group, while there was a significant difference in Roseburia among the three groups.

A comparative analysis of KEGG metabolic pathways between the mealworms group and capers-50% group revealed a specific enrichment in apoptosis-related pathways at the third level (Figure 4E). A differential analysis of KEGG metabolic pathways between the mealworms group and capers-80% group revealed that, at the second and third levels, the capers-80% group was consistently enriched in pathways dedicated to biosynthesis and lipid metabolism. Conversely, the mealworms group showed significant enrichment in pathways supporting substrate digestion and carbohydrate metabolism (*p* < 0.05) (Figures 4F,G).

### 3.4 Differential analysis and functional annotation of fecal metabolites in *Teratoscincus roborowskii* fed mealworm or capers-based diets

**Figure 5:**
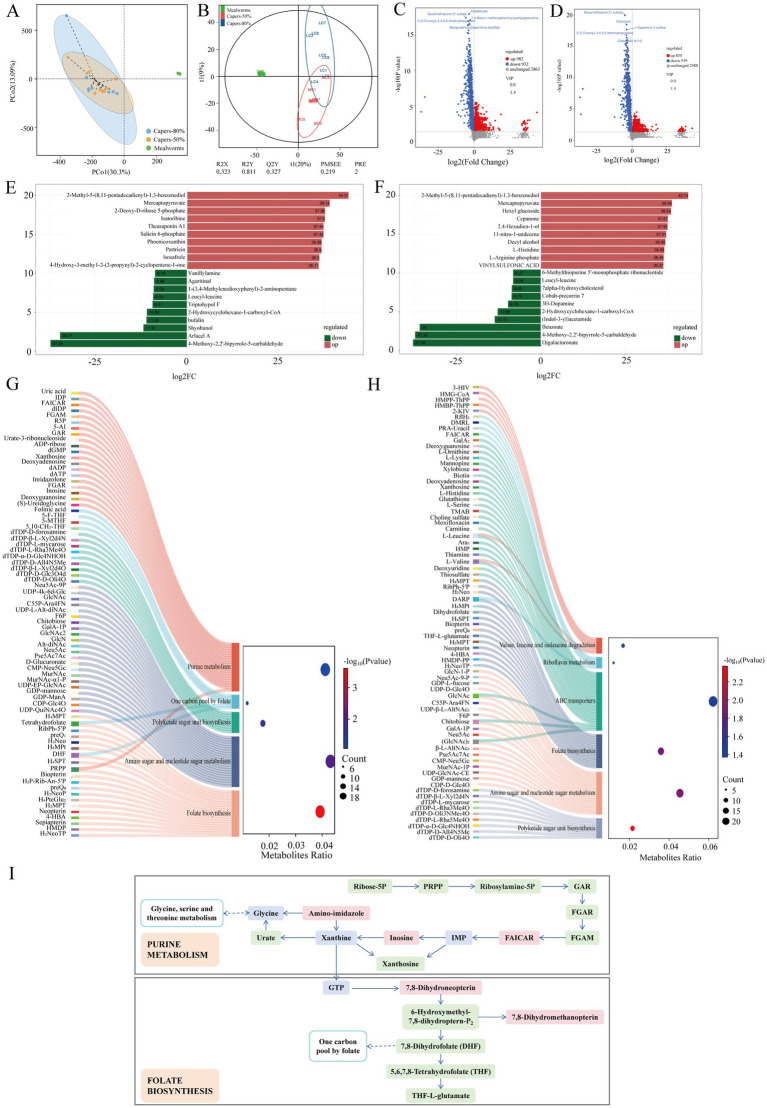
Differential metabolites and KEGG function prediction in *T. roborowskii* fed mealworm or capers-based diets. PCoA **(A)** and PLS-DA analysis **(B)** of fecal metabolites in *T. roborowskii* fed mealworm or capers-based diets. Volcanic maps of different metabolites in the capers-50% group **(C)** and capers-80% group **(D)** compared with the mealworms group. Fold change analysis of metabolites in the capers-50% group **(E)** and capers-80% group **(F)** compared with the mealworms group. Enrichment of functional pathways corresponding to differential metabolites in the capers-50% group **(G)** and capers-80% group **(H)** compared with the mealworms group. **(I)** Purine metabolism and folate biosynthesis pathways, while the red and green blocks indicate the differential metabolites involved in these pathways.

Based on the PcoA analysis, a superior separation was observed between the mealworms and capers groups (Figure 5A). The PLS-DA model revealed that the fecal metabolites of the mealworms and capers groups could be significantly distinguished (Figure 5B).

According to the criteria of *p* < 0.05, VIP > 1, and FC > 1, 1914 differential metabolites were identified. In the capers-50% group, 982 metabolites were upregulated, while 932 metabolites were downregulated. Specifically, compared with the mealworms group, the levels of carbamazepine-o-quinone, 4-Carbamimidoyl-L-phenylalanine, 4-Amino-5-hydroxymethyl-2-methylpyrimidine, and others were significantly increased, while the levels of dialdehyde, dexamethasone 21-sulfate, 1,4-Bis((m-methoxyphenoxy)acetyl)piperazine, and others were significantly increased in the mealworms group (Figure 5C). According to the criteria of *p* < 0.05, VIP > 1, and FC > 1, 1789 differential metabolites were identified. In the capers-80% group, 850 metabolites were upregulated, while 939 metabolites were downregulated (Figure 5D). The absolute values of log_2_FC (Fold Change, FC) were sorted to obtain the top 10 metabolites in each group. In the mealworms group, the contents of metabolites such as 4-Methoxy-2,2′-bipyrrole-5-carbaldehyde, arlacel A, shyobunol, and others were relatively higher. 2-Methyl-5-(8,11-pentadecadienyl)-1,3-benzenediol, mercaptopyruvate, 2-Deoxy-D-ribose 5-phosphate, and others were more abundant in the capers-50% group (Figure 5E). In the mealworms group, the contents of metabolites such as digalacturonate, 4-Methoxy-2,2′-bipyrrole-5-carbaldehyde, benzoate, and others were relatively higher. 2-Methyl-5-(8,11-pentadecadienyl)-1,3- benzenediol, mercaptopyruvate, hexyl glucoside, and others were more abundant in the mealworms group (Figure 5F).

The Sankey-bubble plots showed the enrichment information of different metabolites of *T. roborowskii* in key differential pathways between the capers group and mealworms group. The bubble diagram shows the metabolic pathways enriched by different metabolites of *T. roborowskii* in the capers-50% group and mealworms group include amino sugar and nucleotide sugar metabolism, purine metabolism etc., which were significantly enriched in both groups. The KEGG enrichment network diagram shows that these differential functional pathways are closely related to the differential metabolites between the two groups. Specifically, in the capers-50% group and the mealworms group, tetrahydrofolate, DHF, and PRPP correspond to multiple metabolic pathways (Figure 5G). The metabolites of the capers-80% group and mealworms group were differentially enriched in metabolic pathways such as polyketide sugar unit biosynthesis, amino sugar and nucleotide sugar metabolism, folate biosynthesis, etc. In the capers-80% group and the mealworms group, L-Leucine, L-Valine, DARP, GlcNAc, chitobiose, and (GlcNAc)_2_ also correspond to multiple metabolic pathways (Figure 5H). Furthermore, we found that the main metabolic pathways enriched in the capers group were purine metabolism and folate biosynthesis, and their differential metabolites were also significantly enriched. The metabolic pathways of purine metabolism and folate biosynthesis based on differential metabolites were shown in Figure 5I.

### 3.5 Microbiota-metabolome association in *Teratoscincus roborowskii* fed mealworm or capers-based diets

**Figure 6:**
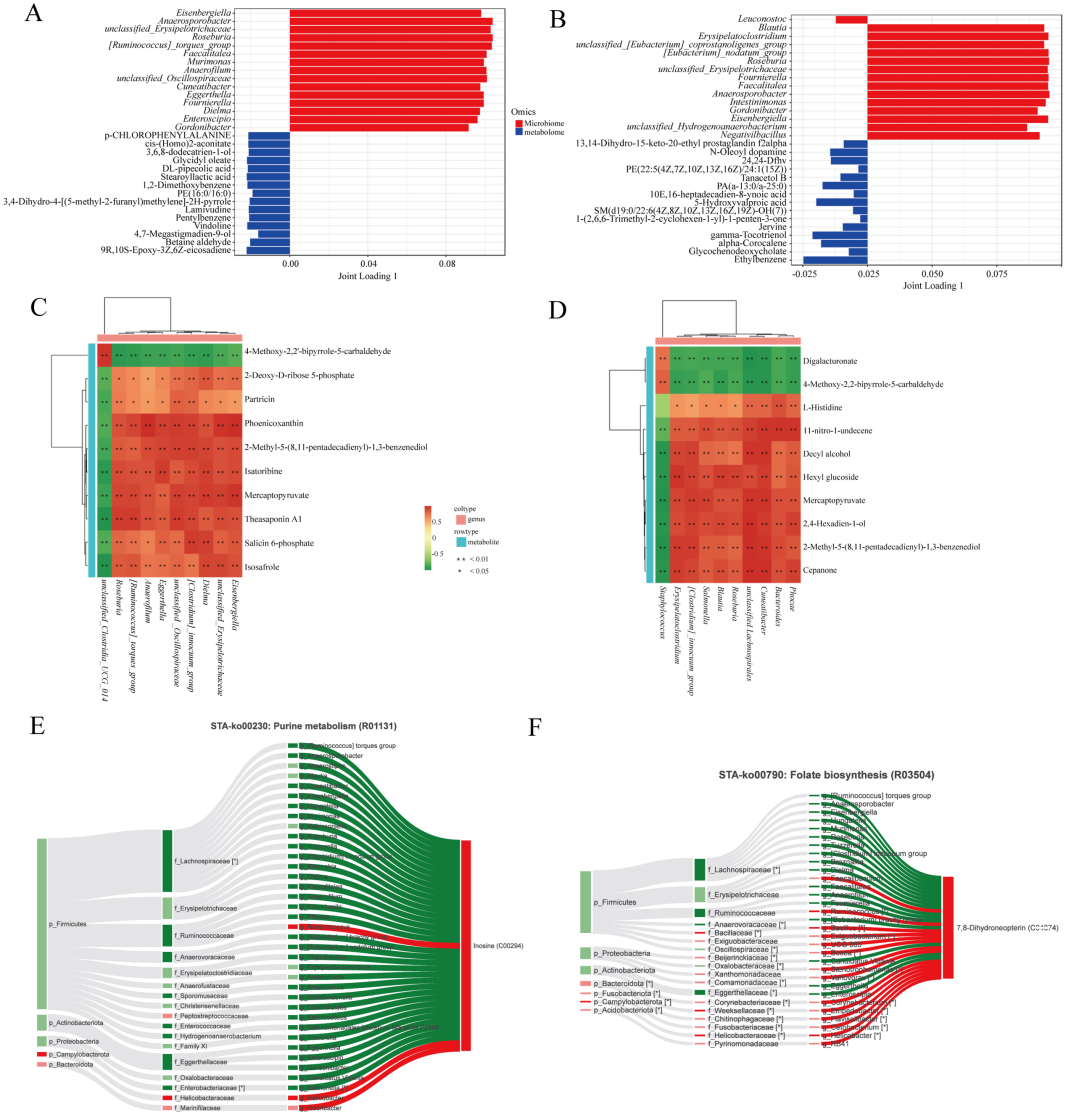
Correlation analysis between gut microbiota and metabolites. **(A)** Load plot of gut microbiota associated with metabolites in the mealworms and capers-50% groups. **(B)** Load plot of gut microbiota associated with metabolites in the mealworms and capers-80% groups. **(C)** Correlation analysis between differential genera and top 10 differential metabolites in mealworms and capers-50% groups. **(D)** Correlation analysis between differential genera and top 10 differential metabolites in the mealworms and capers-80% groups. ^*^p < 0.05. ^**^p < 0.01. (E, F) STA-Sankey network of C00294 for purine metabolism **(E)** and C04874 for folate biosynthesis **(F)** pathways of the mealworms and capers-50% groups.

A bar chart was created to illustrate the top 15 metabolites and microbes ranked by the length of their loading values, representing the highest degree of association. In the capers-50% group, microorganisms such as *Anaerococcus*, *Roseburia*, and *Faecalitalea* show a strong correlation with metabolites (Figure 6A). Additionally, microorganisms such as *Blautia*, *Roseburia*, and *Faecalitalea* show a strong correlation with metabolites in the capers-80% group (Figure 6B).

Correlation heatmaps were drawn to explore the relationship between differential metabolites (top10) and differential microbiota (10 genera). The differential microbiota were closely related to the differential metabolites, showing different degrees of positive or negative correlation. In the mealworms group and capers-50% group, *[Ruminococcus]_torques_group*, *Roseburia*, and others had a significant positive correlation with mercaptopyruvate, isatoribine, and others, and a significant negative correlation with 4-Methoxy-2,2′-bipyrrole-5-carbaldehyde (Figure 6C). In the mealworms group and capers-80% group, *Staphylococcus* had a significant positive correlation with digalacturonate and 4-Methoxy-2,2-bipyrrole-5- carbaldehyde, and a significant negative correlation with cepanone, mercaptopyruvate, hexyl glucoside, etc. *Bacteroides*, *Roseburia*, *Blautia*, and others had a significant positive correlation with cepanone, L-Histidine, and others, while a significant negative correlation with digalacturonate and 4-Methoxy-2,2-bipyrrole-5- carbaldehyde (Figure 6D).

The STA-Sankey networks and Spearman correlation analysis were conducted between microbiota and metabolites in purine metabolism and folate biosynthesis. The results indicated that the key differential metabolite in purine metabolism was inosine, which was positively correlated with *Ruminococcus*, *Helicobacter*, and *Odoribacter* (Figure 6E). The key differential metabolite in folate biosynthesis was 7,8-Dihydroneopterin, which was positively correlated with *Ruminococcus*, *Bacillus*, *Corynebacterium*, *Helicobacter*, and others (Figure 6F).

### 3.6 Correlation analysis of nutrition, gut microbiota, metabolites, and digestive enzyme activities in *Teratoscincus roborowskii* fed mealworm or capers-based diets

**Figure 7:**
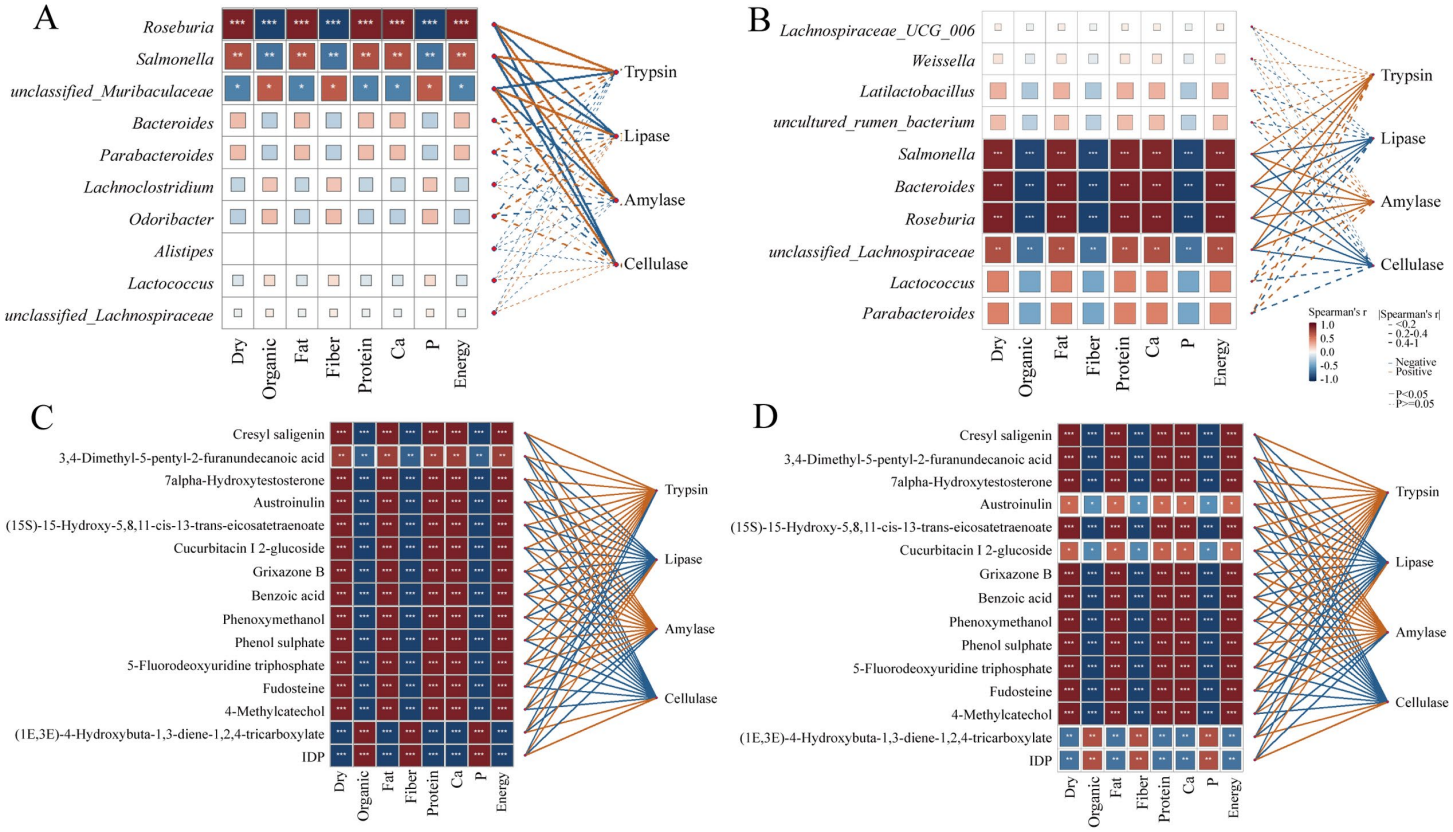
Correlation analysis of nutrition, differential gut microbiota, metabolites, and digestive enzyme activities in *T. roborowskii* fed mealworm or capers-based diets. **(A)** Correlation heatmap of different gut microbiota, nutrition and digestive enzyme activities in the mealworms and capers-50% groups. **(B)** Correlation heatmap of different gut microbiota, nutrition and digestive enzyme activities in the mealworms and capers-80% groups. **(C)** Correlation heatmap of different metabolites, nutrition and digestive enzyme activities in the mealworms and capers-50% groups. **(D)** Correlation heatmap of different metabolites, nutrition and digestive enzyme activities in the mealworms and capers-80% groups.

The correlations among nutrition, digestive enzyme activities, differential metabolites, and differential microbiota were analyzed for the mealworms and capers-50% groups, as well as the mealworms and capers-80% groups. Nutrition was closely related to the differential metabolites, and digestive enzyme activities had different positive or negative correlations with the abundance of different microbiota. Between the mealworms and capers-50% groups, *Roseburia* and *Salmonella* had a significant positive correlation with fat, protein, calcium, and energy, and a significant negative correlation with organic matter, fiber, and phosphorus. In addition, *unclassified_Muribaculaceae* had a significant positive correlation with organic matter, fiber, and phosphorus. Notably, the abundance of *Roseburia* and *Salmonella* was significantly positively correlated with trypsin and α-amylase. However, their abundance was significantly negatively correlated with lipase and cellulase. The abundance of *unclassified_Muribaculaceae* was significantly positively correlated with lipase and cellulase, and significantly negatively correlated with trypsin and α-amylase (Figure 7A). Between the mealworms and capers-80% groups, *Roseburia*, *Bacteroides*, and *Salmonella* had a significant positive correlation with fat, protein, calcium, and energy, and a significant negative correlation with organic matter, fiber, and phosphorus. Notably, the abundance of *Roseburia*, *Salmonella*, and *Bacteroides* was significantly positively correlated with trypsin and α-amylase. However, their abundance was significantly negatively correlated with lipase and cellulase (Figure 7B). Between the mealworms group and the capers groups, 3,4-Dimethyl- 5-pentyl-2-furanundecanoic acid, 7alpha-Hydroxytestosterone, austroinulin, and benzoic acid, and others demonstrated significant positive correlations with fat, protein, calcium, and energy. In addition, these metabolites were positively correlated with trypsin and α-amylase (Figures 7C,D).

## 4 Discussion

*T. roborowskii* is an endemic species with distinctive frugivorous behavior, including the consumption of capers (Gao et al., 2023). After feeding on a grape-rich diet, this species displayed a notable shift in gut microbiota composition, particularly marked by an increase in the probiotic *Lactococcus* (Wang et al., 2024a,b)*. T. roborowskii* in traditional captivity were provided with free access to mealworms, vitamin water, and calcium powder. In this study, however, a caper-based diet was introduced, which increased the intake of plant fiber, water, and other nutrients. Our results demonstrated that the addition of capers significantly increased the abundance of *Blautia* in *T. roborowskii*, enhancing the folate biosynthesis and purine metabolism pathways, confirming that changes in diet can lead to rapid shifts in the gut microbiota composition.

### 4.1 Dietary nutrient composition and digestive enzyme activity

Animals can better adapt to variations in dietary nutrient content and food availability in the environment by modulating their digestive systems accordingly (Karasov and Douglas, 2013). Carnivorous fish exhibit the highest protease activity, followed by omnivorous fish, while herbivorous fish exhibit the lowest levels. In contrast, amylase activity exhibites the inverse profile of protease activity. Lipase activity is also generally higher in herbivorous and omnivorous fish than in carnivorous species (Jiao et al., 2023). Fish with herbivorous diets exhibit higher lipase activity levels than carnivores or omnivores. This difference may reflect lipid scavenging by fish consuming low-lipid foods (Leigh et al., 2018). Herbivorous diets are generally high in fiber, which binds fat in the digestive tract and thereby reduces lipid digestibility (German et al., 1996). Furthermore, when low-protein diets are consumed by herbivores, lipids may be utilized as a protein-sparing energy source, conserving the assimilated protein for tissue maintenance (Watanabe, 1982; German et al., 2004).

In *Podarcis siculus*, omnivorous populations that consume more plant matter display significantly higher amylase activity than strictly insectivorous populations (Wehrle et al., 2020). Increased digestive substrate concentration requires increases in matched enzyme activities to achieve high digestibility of the nutrient. However, intestinal amylase activity was observed to decline as dietary crude fiber levels increased (Wang et al., 2022). In our study, enzyme activity patterns were consistent with nutritional composition analysis results. We observed significantly higher α-amylase activity in the capers-50% group compared to the mealworms group, while lipase activity was significantly higher in the capers-80% group than in the mealworm and capers-50% groups. This suggests that higher lipase activity may be necessary in the capers-80% group to break down the fat content in the diet. It is suggested that high dietary fiber increases the digesta volume and accelerates intestinal transit, resulting in reduced enzyme activity by limiting the time for enzyme-substrate interactions. Trypsin activity was higher in the mealworm group, aligning with the higher crude protein content of that diet. Although cellulase activity did not differ significantly across the three groups, we observed an upward trend in cellulase activity after capers consumption, which was consistent with the higher coarse fiber content in the caper group. Those findings support the idea that there is an alignment between diet and digestive physiological functions in animals. Therefore, *T. roborowskii* may rapidly regulate its digestive physiology to adapt to dietary shifts.

### 4.2 Gut microbiome analysis

Diets with greater diversity tend to support a more diverse gut microbiota (Amamoto et al., 2022). For example, Var*anus salvator* fed with a varied diet of bullfrogs, eggs, and depilated chicken exhibited higher alpha diversity in its gut microbiota compared to wild individuals (Du et al., 2022). However, the complexity of the gut microbiota does not necessarily increase with dietary variety, because different foods contain chemical elements that can either promote or inhibit the growth of specific microbial groups (Li et al., 2016). In our study, no significant differences in alpha diversity were observed among the three groups at the genus level. However, at the phylum level, the Chao1 index was significantly higher in the capers-80% and capers-50% groups compared to the mealworms group. In natural environments, individuals with similar diets tend to share similar gut microbial compositions (Graf et al., 2015). Our findings indicate that short-term caper-based diet significantly altered the beta diversity of *T. roborowskii’*s gut microbiota. The distribution within the group in the PcoA plots was more discrete in the caper groups and more uniform in the mealworms group, suggesting that caper intake influenced the aggregation and stability of the gut microbiota in captive *T. roborowskii*.

Firmicutes and Bacteroidetes are the predominant bacterial phyla that inhabit many organisms. Firmicutes are known for their strong capacity to depolymerize dietary fibers. They can depolymerize different types of dietary fibers and produce metabolites such as butyric acid or lactic acid, which are beneficial to human health (Sun et al., 2023). Bacteroidetes are primary degraders of polysaccharides. Within the Bacteroidetes genome, carbohydrate-degrading enzymes (CAZymes) are arranged in gene clusters known as polysaccharide utilization loci (PULs) (Lapébie et al., 2019). In our study, the dominant microbiota of *T. roborowskii* consisted of Firmicutes and Bacteroidetes at the phylum level, which is consistent with the results of previous findings (Zhang et al., 2022). As gut commensals, *Bacteroides* play multiple roles; they provide protection against pathogens and supply nutrients to other microbial residents of the gut (Zafar and Saier Jr, 2021). *Lactococcus* is a widely recognized probiotic microorganism that helps regulate the intestinal microbial balance in animals and supports host immune performance (Wu et al., 2023). The dominant genera in the capers-50% group were *Bacteroides* and *Lactococcus*, while *Weissella* and *Lactococcus* dominated in the capers-80% group. The probiotic potential of *Weissella* strains is linked to their remarkable ability to survive passage through the gastrointestinal tract, produce antimicrobial substances against various pathogens, and promote the formation of gut-stimulating metabolites (Teixeira et al., 2021). *Bifidobacteria* are also widely used as probiotics due to their beneficial effects on the host’s digestive, immune, and nervous systems (He et al., 2023). We speculate that a certain proportion of capers may act as prebiotics, promoting the enrichment of probiotics. In captivity, constant cohabitation, social interaction, and interaction with human keepers increase opportunities for microbial transmission from host-associated sources. This, consequently, may contribute to an increased microbiota richness of captive animals (Nelson et al., 2013). Our results show that *Salmonella* was the dominant bacterium in the mealworms group. *Salmonella* is a foodborne pathogen that predominantly resides in the intestinal tract of humans and animals. *Salmonella* infections can lead to a range of illnesses, including gastroenteritis, bacteremia, septicemia, and focal infections, with severe cases potentially resulting in host death (Lu et al., 2025). Therefore, dietary diversification through the addition of capers may restructure the gut microbiota to reduce the dominance of potential pathogens and support gut health.

*Blautia* is a genus of anaerobic bacteria with probiotic characteristics commonly found in the feces and intestines of mammals (Liu et al., 2021). It plays a role in various metabolic and inflammatory diseases, as well as in biotransformation processes (Inoue et al., 2017; Kim et al., 2014; Wu et al., 2016). Most of its beneficial properties are linked to its potential probiotic functions, such as the production of bacteriocins, which have the potential to inhibit the colonization of pathogenic bacteria in the intestine and affect the composition of intestinal microbiota (Kim et al., 2019). LEfSe analysis showed a significant increase in *Blautia* abundance in the capers-50% group, suggesting that capers are beneficial dietary components for *T. roborowskii*. Recent studies have reported alterations in the abundance of *Alistipes* in human patients and mouse disease models. *Alistipes* dysbiosis can have either beneficial or harmful effects. It has been associated with liver fibrosis (Rau et al., 2018), colorectal cancer (Moschen et al., 2016), cardiovascular disease (Zuo et al., 2019), and mood disorders (Bangsgaard Bendtsen et al., 2012), among other potential diseases. Additionally, putrefaction, a unique method of fermenting amino acids, implicates *Alistipes* in playing a critical role in inflammation and disease (Parker et al., 2020). However, oral administration of *Alistipes shahii* As360 has been shown to alleviate the symptoms of colitis, modulate the release of cellular inflammatory factors, protect the intestinal epithelial barrier, and alters gut microbiota and fecal metabolites (Lin et al., 2025). In our study, LEfSe analysis revealed a marked increase in the relative abundance of *Alistipes* in the capers-50% group, highlighting the potential benefits of incorporating capers as a dietary component of *T. roborowskii*. Capers contain a variety of biologically active compounds, including polysaccharides, alkaloids, glycosides, tannins, phenolic compounds, and flavonoids, which possess a range of pharmacological functions (Zhang and Ma, 2018), such as antioxidant (Sonmezdag et al., 2019), anti-inflammatory (El Azhary et al., 2017), antitumor (Moghadamnia et al., 2019), antiarthritic (Feng et al., 2011), and antidiabetic activities (Kazemian et al., 2015). Previous research has indicated that capers possess inhibitory effects against ulcerative colitis, modulate gut microbiota, and function as potential prebiotics (Zhu et al., 2021). Flavonoids and their metabolites can shape the gut microbiota by inhibiting the growth of various pathogens and promoting beneficial genera, such as *Bifidobacterium* and *Lactobacillus*. These effects can improve gut health by reducing endotoxin production, enhancing the conversion of primary to secondary bile acids, maintaining gut immune homeostasis, and promoting nutrient absorption (Pei et al., 2020). Flavonoids can also directly interact with the microbiota, leading to changes in microbiota profiles, such as beneficial bacterial growth (Oteiza et al., 2018). Therefore, we speculate that the widespread emergence of probiotics may be related to caper intake. Differential KEGG metabolic pathways analysis between the mealworms and capers-80% groups revealed that at the second hierarchical level, lipid metabolism pathways were significantly enriched in the capers-80% group. At the third level, both fatty acid metabolism and fatty acid biosynthesis were significantly enriched in this group, consistent with the nutrition characteristics of the caper diet. Therefore, these changes may be related to the consumption of capers by *T. roborowskii*.

### 4.3 Metabolome analysis

The addition of different types of diets can lead to metabolic changes. Based on the PcoA analysis, a distinct separation was observed between the mealworms and capers groups, which revealed a remarkable alteration in the metabolites of the *T. roborowskii*. The PLS-DA model demonstrated that the fecal metabolites of the mealworms and capers groups could be significantly distinguished, indicating distinct metabolic profiles between the two groups. Mercaptopyruvate is a substrate of 3-mercaptopyruvate sulfur transferase (MPST). MPST has been reported to mediate direct protein-to-protein transpersulfidation reactions beyond its previously known protein substrates thioredoxin and MOCS3/Uba4, which are associated with H_2_S generation and tRNA thiolation, respectively (Pedre et al., 2023). 4-Amino-5-hydroxymethyl-2-methylpyrimidine is an intermediate in thiamin (vitamin B1) synthesis. Thiamin is an essential nutrient for cellular metabolism. Microorganisms that are unable to synthesize thiamine, either fully or in part, obtain it exogenously from their environment or through interactions with other microbes in their community (Sathe et al., 2022). Thiamin participates in both the synthesis and breakdown of purine nucleotides, thereby promoting uric acid metabolism and excretion. Purine metabolism plays several important roles in cells, particularly in DNA and RNA synthesis. Uric acid is the end product of purine metabolism. The gut microbiota contributes to the symbiotic cycle of adenine metabolites (Chiaro et al., 2017) and produces and release significant amounts of purines (Lee et al., 2020). The accumulation of purine degradation intermediates, such as inosine, provides ample substrates for the salvage pathway, that characterized by a lower energy cost and thus contributes to energy conservation, in comparion with *de novo* synthesis (Tran et al., 2024).

*Bacteroides thetaiotaomicron* can increase gut-liver folate levels, which alleviates oxidative stress, supports DNA repair, and maintains metabolic homeostasis (Li et al., 2024; Woo et al., 2008; Boonma et al., 2022; Li et al., 2022). Our metabolomics revealed that capers enhance purine metabolism in *T. roborowskii*, as shown by increased 4-Amino-5-hydroxymethyl-2-methylpyrimidine. Xanthine, a product of purine metabolism, can be converted into guanosine triphosphate (GTP), which serves as a precursor for the folate biosynthesis pathway. Thus, the capers’ enhancement of purine metabolism and *Bacteroides* may promote folate biosynthesis, thereby potentially improving the survival capacity of *T. roborowskii* in desert environments. Consequently, capers appear to play a beneficial role in the diet of *T. roborowskii*. In future studies, we will isolate the active components of capers, such as flavonoids, and elucidate their direct effects and mechanisms on key gut microbiota.

### 4.4 Correlation analysis

Microbiome and metabolomic data can reveal potential links between microbial metabolites and various disease states (Feng et al., 2016). L-Histidine (HIS) is a nutritionally essential amino acid with unique biochemical and physiological properties. HIS and HIS-containing dipeptides can be used to treat metabolic syndromes, atopic dermatitis, ulcers, and inflammatory bowel diseases (Holeček, 2020). According to the correlation heatmap, *Bacteroides*, *Roseburia*, and *Blautia* showed significant positive correlations with L-histidine. This correlation suggests a potential link between changes in the gut microbiota composition under capers feeding and alterations in host histidine metabolism. Correlation analysis between metabolites and the gut microbiota revealed that certain specific bacterial groups respond rapidly to changes in food composition. *Roseburia* is a commensal bacterium that produces short-chain fatty acids, particularly butyrate, which plays a role in colonic motility, immunity maintenance, and exhibits anti-inflammatory properties (Tamanai-Shacoori et al., 2017). Notably, the correlation analysis between differential microorganisms and differential metabolites in the caper group showed that *Roseburia* showed a positive correlation with the majority of differential metabolites. Its increased abundance in the caper groups compared to the mealworms group suggests that the addition of capers to the diet may be associated with a beneficial modulation on the gut microbial environment of *T. roborowskii*.

Inosine, a key secondary metabolite in purine metabolism, acts as a molecular messenger in cell signaling pathways. It is found in various nucleic acids and plays an important role in neuronal signaling (Srinivasan et al., 2021). 7,8-dihydroneopterin is a potent, non-selective antioxidant synthesized by macrophages in response to γ-interferon during immune activation (Wachter et al., 1989). Extensive evidence has demonstrated its effectiveness in scavenging oxidants and free radicals, protecting against oxidative modification of proteins, lipids, DNA, and macrophages during inflammation (Gieseg et al., 2008). *Ruminococcus* species play important roles in the gut ecosystem by degrating plant polysaccharides and participating in hydrogen transfer. This is of great significance for host health and biotechnological applications (La Reau and Suen, 2018). In patients with hyperuricemia, elevated levels of *Ruminococcus torques* and *Ruminococcus gnavus* were accompanied by increased levels of uric acid precursors such as inosine and xanthine (Fu et al., 2024). This suggests that *Ruminococcus* can influence host purine metabolism. Interestingly, *Ruminococcus flavefaciens* strains 1,607 and 1,625 differ from many *Ruminococci* previously studied in that they do not require either p-aminobenzoic acid or folic acid, but require tetrahydrofolate for maximum growth (Slyter and Weaver, 1977). Differential metabolites from the capers-50% and capers-80% groups were compared with those from the mealworms group, and their associations with gut microbiota were visualized using STA-Sankey networks. The key differential metabolite in purine metabolism was inosine, which positively correlated with *Ruminococcus*, *Helicobacter*, and *Odoribacter*. The key differential metabolite in folate biosynthesis was 7,8-dihydroneopterin, which was positively correlated with *Ruminococcus*, *Bacillus*, *Corynebacterium*, among others. These results suggest that *Ruminococcus* may be associated with folate biosynthesis.

A large proportion of digestive enzymes in the intestine originate from the gut microbiota. Most *Bacteroides* live in the distal gut and can digest dietary and host-derived polysaccharides. By fermenting these polysaccharides into short-chain volatile fatty acids, they provide nutrition to the host, maintain the stability of the intestinal microecology, and profoundly impact the host immune system (Hao et al., 2021). Correlation analysis between digestive enzymes, gut microbiota, and nutritional factors indicated that *Roseburia* and *Bacteroides* showed a significant positive correlation with fat, proteins, and energy. The positive correlations of these bacteria with fat, protein, and energy suggest their potential involvement in the metabolic processes of these nutrients, which could lead to enhanced breakdown and energy release. Bacteroidetes are known to produce abundant polysaccharide-degrading enzymes, such as amylase (Brown et al., 2023). The abundance of *Roseburia* and *Bacteroides* was significantly positively correlated with trypsin and α-amylase. Thus, the high abundance of Bacteroidetes was strongly associated with the higher α-amylase activity, implying a potential contributory role. *Muribaculaceae* can produce short-chain fatty acids and regulate intestinal barrier function and immune responses, and are considered promising “next-generation probiotics.” These bacteria utilize dietary fiber as an energy source, which supports their colonization of the gut (Zhu et al., 2024). *Unclassified Muribaculaceae* showed significant positive correlations with organic matter, fiber, and phosphorus, which in turn were positively correlated with lipase and cellulase. These findings underscore that diet, gut microbiota, and digestive enzymes are closely linked and interact with each other. Although there is no evidence to prove whether changes in digestive enzyme activity are directly caused by these bacteria, they appear to result from host adaptation to dietary changes.

3,4-Dimethyl-5-pentyl-2-furanundecanoic acid is a potent antioxidant and free radical scavenger, allowing it to play an important role in preventing lipid peroxidation and protecting polyunsaturated fatty acids. This compund is involved in fatty acid and lipid metabolic pathways. 3,4-Dimethyl-5-pentyl-2-furanundecanoic acid, 7alpha-Hydroxytestosterone, austroinulin, and benzoic acid showed significant positive correlations with fat, protein, calcium, and energy. In addition, these metabolites were positively correlated with trypsin and α-amylase levels. A species composition of the gut microbiota responds to dietary changes, and metabolic outputs of the microbiota are influenced by the supply of dietary components and diet-mediated changes in microbiota composition. The breakdown of substrates by digestive enzymes in the gut also affects metabolite production. Consequently, diet-induced adaptive responses in intestinal function appear to result from complex interactions among diet, microbiota, associated metabolites, and digestive enzymes.

## 5 Conclusion

**Figure 8:**
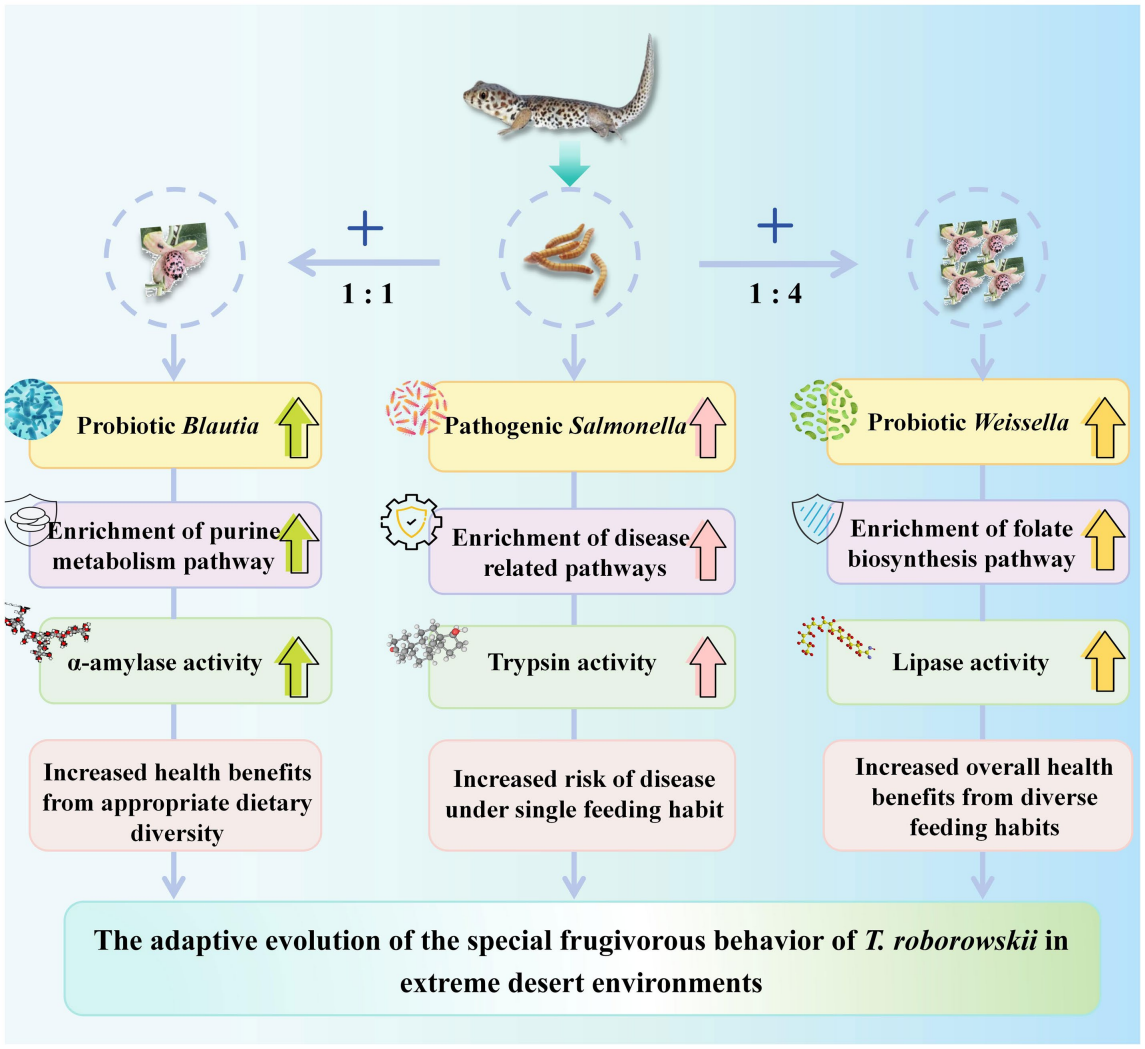
Illustration of different diets on gut microbiota and digestive physiology in *T. roborowskii*.

In summary, the addition of capers to the diet caused significant changes in the gut microbiota of *T. roborowskii*. Our results indicate that caper consumption caused a notable shift in the microbiota composition, in particular, an increase in beneficial bacteria such as *Blautia*. As a result, metabolic pathways related to purine metabolism and folate biosynthesis were significantly enriched in the caper groups. Additionally, the intake of capers increased the activity of amylase and lipase in *T. roborowskii*, supporting the idea that there is a functional match between the animal’s intestinal function and dietary composition (Figure 8). Our study provides an important supplement to the study of the gut microbiota of desert lizards, and will be worth for further exploring the interaction between desert lizard and desert plants.

## Data Availability

The data supporting the findings of this study are publicly available from the National Center for Biotechnology Information (NCBI) Sequence Read Archive (SRA) at https://www.ncbi.nlm.nih.gov/sra/PRJNA1282615, accession number PRJNA1282615.

## Footnotes

1
http://www.biocloud.net/

2
https://www.biocloud.net
